# Discrimination of Minced Mutton Adulteration Based on Sized-Adaptive Online NIRS Information and 2D Conventional Neural Network

**DOI:** 10.3390/foods11192977

**Published:** 2022-09-23

**Authors:** Zongxiu Bai, Jianfeng Gu, Rongguang Zhu, Xuedong Yao, Lichao Kang, Jianbing Ge

**Affiliations:** 1 College of Mechanical and Electrical Engineering, Shihezi University, Shihezi 832003, China; 2 Key Laboratory of the Ministry of Agriculture and Rural Affairs, Shihezi University, Shihezi 832003, China; 3 Analysis and Test Center, Xinjiang Academy of Agricultural and Reclamation Science, Shihezi 832003, China

**Keywords:** online NIRS, convolutional neural network, different spectral information, classification, adulterated mutton

## Abstract

Single-probe near-infrared spectroscopy (NIRS) usually uses different spectral information for modelling, but there are few reports about its influence on model performance. Based on sized-adaptive online NIRS information and the 2D conventional neural network (CNN), minced samples of pure mutton, pork, duck, and adulterated mutton with pork/duck were classified in this study. The influence of spectral information, convolution kernel sizes, and classifiers on model performance was separately explored. The results showed that spectral information had a great influence on model accuracy, of which the maximum difference could reach up to 12.06% for the same validation set. The convolution kernel sizes and classifiers had little effect on model accuracy but had significant influence on classification speed. For all datasets, the accuracy of the CNN model with mean spectral information per direction, extreme learning machine (ELM) classifier, and 7 × 7 convolution kernel was higher than 99.56%. Considering the rapidity and practicality, this study provides a fast and accurate method for online classification of adulterated mutton.

## 1. Introduction

Mutton is very popular because of its delicious taste and rich nutrition. However, due to the relatively high price, mutton has become the adulteration object to some illegal merchants [1]. In China, adulterated mutton refers to mutton mixed with low-price meat, such as pork or duck, without declaration [1,2,3]. Food adulteration not only harms consumer rights and interests, but also affects the order of the food market [4,5,6]. In particular, pork adulterated in mutton will undermine ethical beliefs for some ethnic groups. Traditional detection techniques of food adulteration are usually based on deoxyribonucleic acid [7], polymerase chain reaction (PCR) [8], and chromatography [9]. However, these methods have some shortcomings, such as being time-consuming, laborious, or costly. Compared with the above methods, near-infrared spectroscopy (NIRS) has the great potential of being fast, nondestructive, and environment-friendly. Although it is not cheap at the moment, low-cost and miniaturised spectrometers are still being developed with the progress of science and technology, and the NIRS technology has great application potential. In recent years, NIRS has been applied to detect the quality and adulteration of meat [10,11,12,13,14,15].

To collect more representative spectral information by using the single-probe NIRS system, some studies reported that their samples had been scanned many times or collected the multipoint spectra from different directions. Barragan et al. [16] collected four spectra of each sample by scanning four times with a single-probe and used mean spectral information to build the model for authentication of barley-finished beef. Alamprese et al. [17] recorded two spectra of each sample by scanning two times for the identification and quantification of minced beef adulteration with turkey meat. Boiret et al. [18] established a detection model for active components in tablets by using the mean multipoint spectral information in two orthogonal directions. Moreover, Duan et al. [19] proved that the spectral information from different regions of interest could affect the model performance by using hyperspectral imaging technology. Although the NIRS system can collect the multipoint spectral information in different ways, there are no reports about the influence of spectral information on the model performance for meat quality detection. To obtain the multipoint spectral information using the single-probe NIRS and explore the influence of different spectral information on the classification model of minced mutton adulteration, it is necessary to develop an online NIRS system, which can adaptively collect multipoint spectra for each sample, and use different spectral information to establish the classification model of minced mutton adulteration.

As one of the representative deep learning algorithms, the convolutional neural network (CNN) can directly extract representative features from the original data, which avoids the cumbersome operation of the traditional method that requires multiple data pre-processing. In recent years, it had been gradually used in the modelling of NIRS due to its excellent ability of spectral feature extraction, high accuracy, and strong robustness [20,21,22]. To adapt to the relevant operation requirements of the convolution layer, the spectral data vector of each sample was transformed into a two-dimensional (2D) spectral information matrix by constructing a spectral information matrix. Padarian et al. [23] used CNN to extract the deep features contained in 2D spectral information matrixes to predict the soil property and proved that CNN was an effective tool for modelling. However, there are some studies that reported that the size of the convolution kernel and the type of classifier could affect the performance of the CNN model. Chen et al. [24] studied the influence of the size of the convolution kernel on the CNN model and found that the coefficient of determination (R2) for the calibration increased with the size of the convolution kernel. Li et al. [25] optimised CNN models by comparison of different kernel sizes and achieved better classification accuracy with a large convolution kernel size. Su et al. [26] used the classifiers of SVM (support vector machine), LSVM, and Softmax for the identification of wheat leaves, and found that LSVM had the highest classification accuracy and lowest iteration times. Sharma et al. [27] compared the CNN-Softmax, CNN-ELM, and CNN-SVM model for fire detection, and the results showed that the classification accuracy of the CNN-ELM (extreme learning machine) model was 2.7–7.1% higher than that of CNN-Softmax. The above research showed that CNN was an effective qualitative classification model, but the sizes of the convolutional kernel and classifiers had influence on its performance. There are also few studies that have used the CNN model combined with different classifiers to detect food adulteration. Therefore, it is meaningful to establish the classification model of adulterated mutton by using CNN combined with different classifiers, and to explore the influence of different convolution kernels and classifiers on the model performance.

In order to explore the influence of different spectral information and model parameters on NIRS classification of minced mutton adulteration based on CNN, the following was carried out in this study: (1) an online NIRS system was developed, which could adaptively collect the spectra of four points in one run according to the size of the sample; (2) samples of pure mutton, pure pork, pure duck, and adulterated mutton (minced mutton mixed with 10–20–30–40–50% (*w*/*w*) pork/duck) were prepared, and the spectral information of samples from four different directions (45° interval between adjacent directions) were collected; (3) the mean spectral information per direction and of four directions were obtained, and the 1D spectral data of samples were converted to a 2D spectral information matrix; (4) the CNN models with different classifiers (Softmax, ELM, and SVM) were established and compared based on different spectral information, and the influence of different spectral information, convolution kernel sizes, and classifiers on models was explored. This study has certain significance for safeguarding the rights and interests of consumers and promoting the healthy development of the mutton industry.

## 2. Materials and Methods

### 2.1. Sample Preparation

In this study, samples of mutton, pork, and duck were purchased from the local supermarket in Shihezi city, Xinjiang autonomous region. The samples were sent to the laboratory in an insulation box with ice (the temperature was between 0 and 5 °C) and then stored in a refrigerator (the temperature was between 0 and 4 °C). Because the white fascia and fat of meat will bring great influence to the spectrum of point acquisition, the visible fat and skin of the meat was removed before sample preparation to reduce the interference in classification. For preparation of pure meat and adulterated meat samples, the trimmed meat was weighted by an electronic scale (YingHeng, China), and they were minced and mixed by a meat-mincing machine (Joyoung, JYS-A900, China) for 30 s. Then, 30 ± 1 g of minced meat was compacted in a round petri dish (60 mm in diameter × 15 mm in depth × 10 mm in thickness) to 10 mm in thickness while keeping its surface smooth, to ensure the homogenisation of the sample. In order to improve the generalisation performance of the classification model, five kinds of samples were prepared. According to 35 samples of each adulterated proportion (10%, 20%, 30%, 40%, and 50%), 350 (2 × 5 × 35) adulterated mutton samples with duck/pork were prepared, and 35 pure mutton, 35 pure duck, and 35 pure pork samples were also obtained.

### 2.2. Online NIRS System

**Figure 1:**
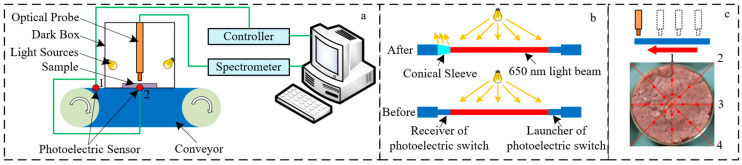
Schematic diagrams of the (**a**) online NIRS system, (**b**) method for avoiding the interference, and (**c**) data acquisition. The input current is converted into an optical signal and emitted by a laser photoelectric switch. The receiver detects the target object according to the intensity of the received light or whether there is no light. A conical sleeve is installed on the receiver of the laser photoelectric switch, which is mainly used to avoid the interference of the light emitted by the light source to the receiver.

The online NIRS system developed in this study mainly includes two pairs of photoelectric sensors, a conveyor, a microprocessor (STM32F103C8T6, STMicroelectronics Inc Geneva, Switzerland,), a NIR spectrometer (900–2500 nm, NIRQuest512, Ocean Optics Inc., Dunedin, USA), two halogen lamps (MR11 20W, Philips Inc., Amsterdam, The Netherlands), a single optical fibre probe (QP400-1-vis-nir, Ocean Optics Inc., Dunedin, USA), a PC machine, and a self-designed software. Its structural schematic diagram is shown in Figure 1a. The speed of the conveyor was set as 8 cm/s according to the preliminary research results of our laboratory and the existing reports on online detection [28,29,30]. The halogen lamps were installed on both sides of the dark box, and the installation angle was set to 30° to the horizontal plane. The vertical distance between the probe and the sample surface was 1.5 cm. The interval time of 4 collection points of each sample was determined by the time interval of the rising and falling edge of the signal from photoelectric sensor 1 (Figure 1a). Photoelectric sensor 2 was used to detect the arrival signal of the sample and send it to the control software, to drive the spectrometer to collect the spectral data of the sample. Due to the interference of light sources on the photoelectric sensor signal, two pairs of 5 V laser sensors with a beam of 650 nm were applied in this system and a conical sleeve was adopted to reduce the influence of light sources, as shown in Figure 1b.

The acquisition software was designed in Qt Creator 4.9.1 (Qt Company Ltd., Finland) by using C++, which was based on the OmniDriver 2.56 (Ocean Optics Inc., Dunedin, FL, USA). The software could complete the parameter configuration of the spectrometer, including integration time, smoothness and scanning times, communication with the microcontroller, and data processing. The online NIRS system developed in this study could adaptively collect four spectra of each sample according to the sample size in the run direction.

### 2.3. Spectral Data Acquisition and Dataset Partition

**Figure 2:**
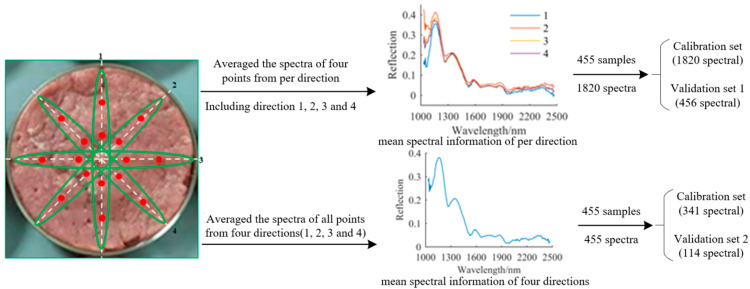
Acquisition process of different spectral information and dataset division.

Before the acquisition of spectra, the NIRS system needed to be preheated about 30 min. In this study, the integral time, smoothness, and scanning times of the NIRS acquisition system were set to 40 ms, 5 times, and 10 times, respectively. The whiteboard (USRS-99-010, Labsphere Inc., Sutton, NH, USA) was used to perform white calibration of the spectrometer, and the light source was turned off for black calibration. The reflection spectra of four points in the run direction of the sample were automatically collected according to the sample size. To study the influence of the spectral information on the classification model, spectra of four directions (1, 2, 3, and 4) at 45° intervals were collected for each sample, as shown in Figure 2. The obtained spectral data were saved as a CSV file for further data processing. A total of 455 samples were used for collecting the spectral information. First, the spectra of four points per direction (including direction 1, 2, 3, and 4) were averaged to obtain the mean spectral information per direction, and a total of 1820 spectra were obtained for 455 samples. Based on the mean spectral information of the four directions, a total of 455 samples were divided into 341 samples for the calibration set and 114 samples for the validation set by the joint x-y distances algorithm, which was first proposed by Galvao et al. [31], and its principle is to calculate the distance between samples by using two variables, label value and spectrum, to ensure the maximum distribution of samples, to effectively cover the multidimensional vector space, to increase the difference and representativeness between samples, and to improve the stability of the model. On this basis, 1820 mean spectra per direction were divided into 1364 spectra for the calibration set and 456 spectra for the validation set. Two kinds of spectral information were used to establish models, and two validation sets from different spectral information were used to test the classification performance.

### 2.4. Model Establishment and Evaluation

**Figure 3:**
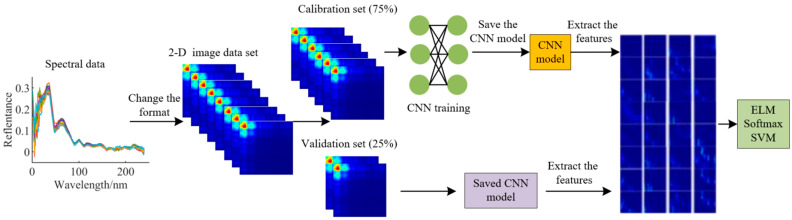
Schematic diagram of data process.

In this study, CNN models with the classifiers of Softmax, ELM, and SVM were established on the basis of a 2D spectral information matrix. The best model for classification of adulterated mutton was selected by comparing the performance of the models. The schematic diagram of the data process is shown in Figure 3.

#### 2.4.1. Modelling Methods

The CNN model usually consists of an input layer, hidden layer, and output layer, and the hidden layer contains a convolution layer, pooling layer, and fully connected layer. The main function of the convolution layer is to extract features of input data [32]. The output data are obtained by multiplying the convolution kernel with the corresponding element values in the coincidence region of input data and adding an offset, and its convolution operation is described as Formula (1):

(1) $$X_{i}^{\left(k\right)}=\sum_{n=1}^{N}W_{i}^{\left(n,k\right)}⊗X_{i-1}^{\left(n\right)}+B_{i}^{k}$$

where $X_{i}^{\left(k\right)}$ is the output of the convolution layer; *i* is the serial number of the convolution layer; *k* is the serial number of the convolution kernel; *N* is the number of channels of input data; *W* and *B* are the weight and offset, respectively; ⊗ represents the convolution operation.

After convolution, the output data are input to the next layer by the nonlinear transformation activation function. The most commonly used activation function is rectified linear unit (ReLu), which is described as Equations (2) and (3) [33]:

(2) $$f(x)=\left\{\begin{array}{c} x,x\geq 0\\ 0,x<0 \end{array}\right.$$

(3) $$f(X_{i}^{\left(k\right)})=f(\sum_{n=1}^{N}W_{i}^{\left(n,k\right)}⊗X_{i-1}^{\left(n\right)}+B_{i}^{k})$$

The function of the pooling layer is reducing dimensions and selecting representative features from input data [34]. The pooling kernel can be set to different sizes, and the output result of the pooling layer is calculated by moving the pooling kernel in the region of the input data matrix according to the stride.

In general, the CNN is composed of two or more fully connected layers, and the neurons of fully connected layers are completely connected between the two layers. Two-dimensional features from the last pooling layer are flattened. Then, it is regarded as the input of the full connection layer for the further feature extraction [35]. By calculating in fully connected layers, a one-dimensional vector is output, and each value of the vectors represents the quantitative value of classifications.

The CNN model in this study contained 3 convolution layers, 3 pooling layers, and 2 fully connected layers. The size of the input layer was 230 × 230 × 1 (width × height × channel). Three convolution kernels of different sizes (3 × 3, 5 × 5, and 7 × 7) were set in the convolution layer to discuss its influences on the models, the padding method was the same padding, the size of step was 1, and the activation function was ReLu. The maximum pooling method of 2 × 2 was used to compress images and extract features. To improve the generalisation ability of the whole network and prevent over fitting, dropout layers were added between adjacent convolution layers, and the drop rate was set to 0.3. In the CNN model, the Adam was selected as the optimiser, the mean square error (MSE) was used to measure the loss value, and the learning rate was set to 0.001. In this study, the number of iterations was set to 500, and the CNN was implemented in Python 3.7.6 using Keras library and the Tensorflow 2.3.0 backend.

#### 2.4.2. Classifiers

The classifiers of Softmax, ELM, and SVM were used in this study. A common classifier used in the CNN model is Softmax, which calculates the probability value of each output channel and selects the channel with the highest probability value as the result of classification. As a kind of supervised machine learning algorithm, SVM can classify samples to the maximum extent by mapping nonlinear data to a high-dimensional space [35]. As a fast-learning algorithm, ELM can initialise input weights and offsets randomly, which has the characteristics of strong generalisation ability and fast calculation speed [35]. To select a fast and accurate model that is suitable for online detection of adulterated mutton, the classification performance of the CNN model with different classifiers was compared. Parameters of different classifiers were optimised to obtain better model performance. For the SVM classifier, the radical basis function (RBF) was set to the kernel function, and parameters (gamma and cost) were searched by the optimisation method of the genetic algorithm (GA). The activation function of the ELM classifier was sigmoid, and 10–200 neurons at 5 intervals were used to search the best number of neurons for hidden layers. When the neurons were set as 175, the best performance of the ELM classifier could be obtained.

#### 2.4.3. Model Evaluation

In the classification model of this paper, the samples were classified into five categories: pure mutton, pure pork, pure duck, adulterated mutton with pork, and adulterated mutton with duck samples. To evaluate the classification ability of models, the classification accuracy (Acc) was applied. In order to obtain a fast classification model, the prediction time was used to measure the efficiency of different classifiers.

## 3. Results and Discussion

### 3.1. Spectral Data Analysis

**Figure 4:**
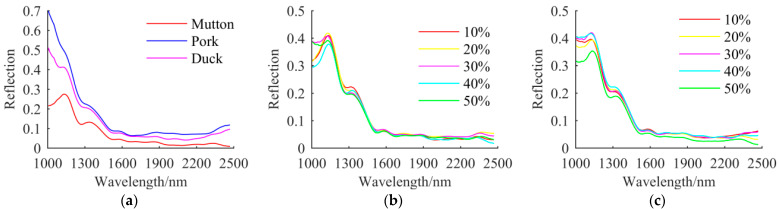
Mean reflectance spectrum of (**a**) pure meat; (**b**) adulterated samples at different percentages with pork; (**c**) adulterated samples at different percentages with duck meat.

To avoid the uninformative bands existing in the NIR spectral data, the range of 1038–2475 nm was selected for modelling, and the number of spectral channels was 230. The Savitzky–Golay (SG) with a five-point filter was applied to smooth the spectra. Figure 4 shows the mean reflectance spectra of representative samples. Figure 4a shows that the spectral trends of mutton, duck, and pork are similar, but there are obvious differences in spectral reflectance because of their different chemical composition [1]. The mean spectrum of pork samples has higher values of reflection than mutton or duck samples. Figure 4b,c show that spectral reflection curves of all samples with different adulteration percentages have similar trends, and the reflectance changes with different adulteration proportions. It is clear that wavelengths around 1260, 1520, 1650, and 1840 nm have significant absorption peaks in the spectral curves of pure meat and adulterated mutton, which are related to the absorption bands of water, fat, and protein in the meat samples. In particular, three absorption peaks at 1260, 1840, and 1650 nm are mainly related to the C-H second overtone and the C-H_2_ stretch first overtone in fat [36], and the absorption peak at 1520 nm is closely related to the N-H stretching second and first overtones in protein [37]. In addition, the range of 1700–2475 nm has a low reflectance value, because there are some absorption bands mainly caused by the combined overtones of molecular groupings such as O-H, N-H, and C-H [38].

To fully utilise the capacity of the CNN model, the 1D spectra were normalised to the range of 0–1 and converted into a 2D spectral information matrix according to Equation (4).

(4) $$S=xx^{T}$$

where *x* is the spectral vector after normalisation; $x^{T}$ is the transposition matrix of *x*; *S* is the matrix of a 2D spectral information matrix.

**Figure 5:**
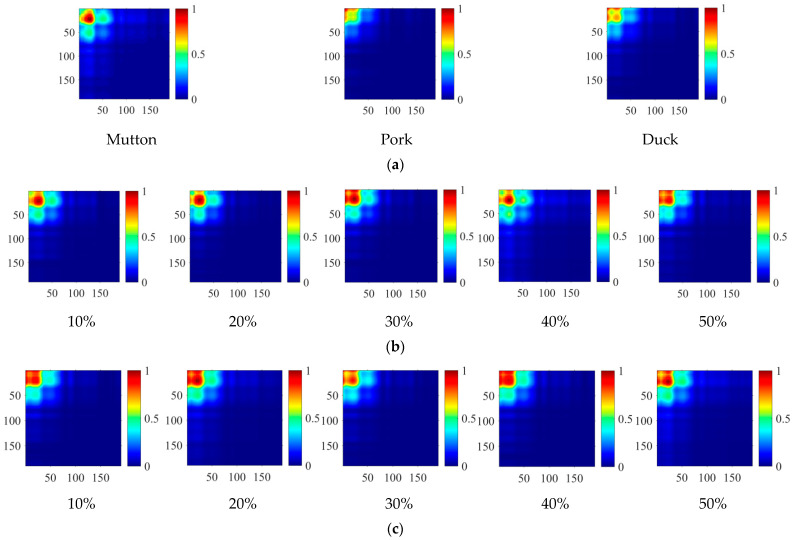
Two-dimensional (2D) spectral information matrixes of (**a**) pure meat; (**b**) adulterated mutton with pork in different proportions; (**c**) adulterated mutton with duck in different proportions.

As the 2D spectral information matrixes converted from the 1D spectral data were single-channel grey images, jet colour was applied to improve the visual recognition ability of spectral information matrix features. Figure 5 shows the mean 2D spectral information matrixes of representative samples. It can be seen that the brightness in the upper left region is larger and decreases along the lower right region. Some differences could be found for different meat. Figure 5a shows that there are brightness values of 0.8–1 at (25, 25), and two brightness values of 0.4–0.6 at (50, 25) and (25, 50) in the mutton spectral information matrixes, while duck and pork samples have no obvious brightness values in the same region. Meanwhile, it can be seen from Figure 5b,c that the spectral information matrixes of adulterated mutton have similar brightness distributions, but there are still slight differences between the spectral information matrixes of samples with different proportions and different types of adulterated mutton. Based on the above differences, CNN can extract different depth features and combine different classifiers to classify different meat.

### 3.2. Model Establishment and Evaluation

The CNN models with different classifiers (Softmax, SVM, and ELM) and convolution kernel sizes (3 × 3, 5 × 5, and 7 × 7) based on two kinds of different spectral information were established, and the model performance was tested with two validation sets from different spectral information. Validation set 1 was the validation set corresponding to the mean spectral information of the four directions; validation set 2 was the validation set corresponding to the mean spectral information per direction. The influence of different mean spectral information, convolution kernel sizes, and classifiers on models was explored, and the optimal models were selected based on the accuracy of the validation set. The high accuracy of the validation set indicated the strong classification ability of the model. When the accuracy of validation sets was the same, the optimal models were selected according to the accuracy of the cross-validation sets, and the high accuracy of the cross-validation set indicated that the model had good stability.

#### 3.2.1. CNN-Softmax Models Establishment Based on Different Spectral Information

**Figure 6:**
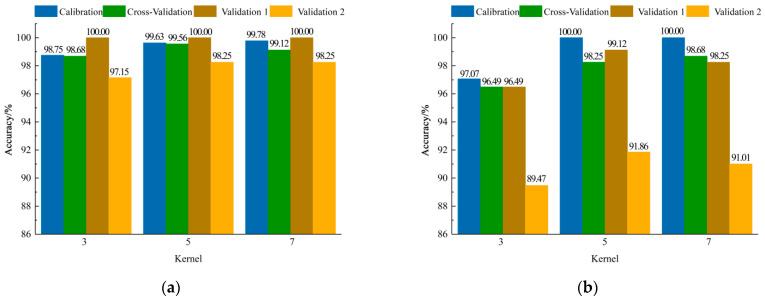
Results of CNN-Softmax models with different convolution kernel sizes based on mean spectral information (**a**) per direction and of (**b**) four directions.

The results of CNN-Softmax models are shown in Figure 6. It could be found that the size of the convolution kernel had little effect on the performance of the models. When the mean spectral information per direction was used to establish the model, the accuracy of all datasets was higher than 97%. By comparing the results of validation sets, the performance of the model with the convolution kernel of 3 × 3 was slightly lower than those with the other two convolution kernels. When the sizes of the convolution kernel were set as 5 × 5 and 7 × 7, the accuracy of validation set 1 and 2 for the two models was the same. By comparing the accuracy of cross-validation sets for the two models, it could be found that the model had the highest accuracy when the size of the convolution kernel was set as 5 × 5, of which the accuracy for validation set 1, validation set 2, and the cross-validation set was 100.00%, 98.25%, and 99.56%, respectively. Similarly, it could be obtained that the accuracy of the model with the size of the convolution kernel of 5 × 5 was highest when the mean spectral information of the four directions was used for modelling, and the accuracy of validation set 1, validation set 2, and the cross-validation set was 99.12%, 91.86%, and 98.25%, respectively.

#### 3.2.2. CNN-SVM Models Establishment Based on Different Spectral Information

**Figure 7:**
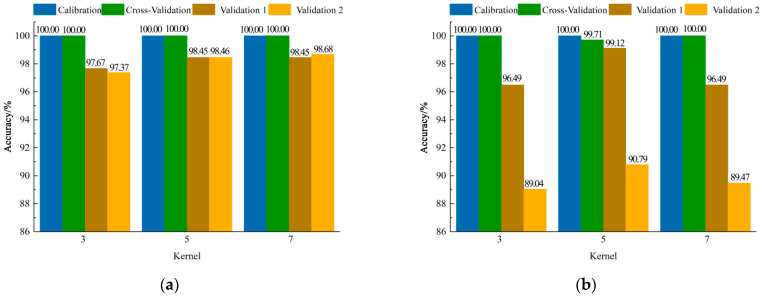
Results of CNN-SVM models with different convolution kernel sizes based on mean spectral information (**a**) per direction and of (**b**) four directions.

Figure 7 shows the results of CNN-SVM models. When the mean spectral information per direction was used for modelling, the accuracy of calibration sets and cross-validation sets for all models was 100.00%. It was found that the classification performance of the model with the 3 × 3 convolution kernel sizes was lowest by comparing the accuracy of validation sets, and when the sizes of the convolution kernel were set as 5 × 5 and 7 × 7, the accuracy of the cross-validation set and validation set 1 had the same results. However, it could be found that the accuracy of the model with the convolution kernel of 7 × 7 was highest by comparing the accuracy of validation set 2, and its accuracy of validation set 1, validation set 2, and the cross-validation set was 98.45%, 98.68%, and 100.00%, respectively. Similarly, the classification accuracy of the model with the convolution kernel size of 5 × 5 based on the mean spectral information of the four directions was highest, and the accuracy of validation set 1, validation set 2, and the cross-validation set was 99.12%, 90.79%, and 99.71%, respectively.

#### 3.2.3. CNN-ELM Models Establishment Based on Different Spectral Information

**Figure 8:**
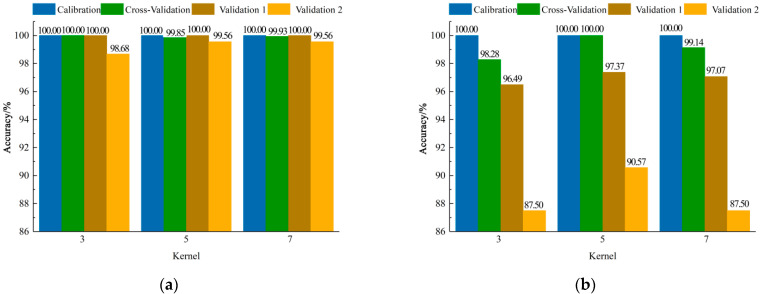
Results of CNN-ELM models with different convolution kernel sizes based on mean spectral information (**a**) per direction and of (**b**) four directions.

Figure 8 shows the results of CNN-ELM models. When the mean spectral information per direction was used to establish the classification model, the accuracy of all models was higher than 98.00%, and the accuracy of validation set 1 for all models was 100.00%. When the size of the convolution kernel was set as 3 × 3, the model performance of validation set 2 was slightly lower than the models with the other two sizes of the convolution kernel. When the size of the convolution kernel was set as 5 × 5 and 7 × 7, the accuracies of the two validation sets for all models were the same; however, it could be found that the model had the highest accuracy when the size of the convolution kernel was set as 7 × 7 by comparing the accuracy of the cross-validation set, and the accuracy of validation set 1, validation set 2, and the cross validation set was 100.00%, 99.56%, and 99.93%, respectively. When modelling with the mean spectral information of the four directions, the classification accuracy was highest when the size of the convolution kernel was set as 5 × 5, and the accuracy of validation set 1, validation set 2, and the cross-validation set was 97.37%, 90.57%, and 100.00%, respectively.

#### 3.2.4. Comparison of the Classification Performance of CNN Models with Different Classifiers

According to the above results, the model established with the mean spectral information per direction was better than the models established with that of the four directions. When two validation sets were used to test the models established by the mean spectral information per direction and of the four directions, it could be found that the models based on the mean spectral information per direction had a higher stability and accuracy than the models based on that of the four directions, and for the same validation set, the maximum difference of accuracy could reach 7.68% for CNN-Softmax, 8.33% for CNN-SVM, and 12.06% for the CNN-ELM model. The results indicated that convolution kernel sizes had little effect on the performance of CNN models, but spectral information had great influence on that, and the models based on the mean spectral information per direction performed better than the models based on that of the four directions. This may be because different average scales have different abilities to retain key information. More effective information is contained in the mean spectra information per direction, so the model has a good effect.

To explore the influence of different classifiers on the models, the optimal models established by different classifiers based on the mean spectral information per direction were compared. The results are shown in Table 1.

Table 1 shows that the accuracy of validation sets for all models based on mean spectral information per direction with the best kernel size was higher than 98.00%. By comparing the accuracy of validation sets 1 and 2, the performance of the CNN-ELM model was better than that of the CNN-Softmax or CNN-SVM model. In order to evaluate the efficiency of the model, the prediction time for validation set 2 of models was calculated. It can be found that the prediction time of the CNN-ELM model was 0.02 s, which is much shorter than those of the other two models. This was because the parameters calculated by a single hidden layer of the CNN-ELM model were less than those calculated by the fully connected layer of the CNN-Softmax model [31]. The above results indicated that classifiers had little effect on model accuracy, but had significant influence on classification speed, and CNN-ELM was more suitable for adulterated mutton detection compared with the CNN-Softmax or CNN-SVM model.

## 4. Conclusions

An online NIRS system based on a photoelectric sensor was developed, which could adaptively collect the spectral information of four points in one run according to the size of the sample. Based on the mean spectral information per direction and of four directions, the CNN models with different classifiers were established to classify samples of pure mutton, pork, duck, and adulterated mutton with pork/duck. The influence of spectral information, the convolution kernel size, and the classifier on model performance was explored. The results showed that the spectral information (mean spectra information per direction and average spectra information of the four directions) had great influence on the classification performance of the model. When the models were tested with two validation sets from different spectral information of the same samples, the models based on the mean spectral information per direction had a higher stability and accuracy than the models based on that of the four directions, and the maximum difference of accuracy could reach 12.06% for the same validation set. In addition, the size of the convolution kernel (3, 5, 7) had little effect on model performance, and classifiers (Softmax, SVM, and ELM) had little effect on model accuracy, but had significant influence on classification speed. The accuracy of all datasets of the CNN-ELM model with spectral information per direction on the basis of the convolution kernel of 7 × 7 was all higher than 99.56%. Considering the rapidity and practicality of online detection, it could meet the fast and accurate online classification of adulterated mutton. The results provide a theoretical basis and technical support for rapid evaluation of adulteration mutton.

## Figures and Tables

**Table 1:** Performance of the CNN-Softmax, CNN-ELM, and CNN-SVM models based on mean spectral information per direction with the best kernel size.

Model	Kernel Size	Calibration Set	Cross-Validation Set	Validation Set 1	Validation Set 2	
		Acc (%)	Acc (%)	Acc (%)	Acc (%)	Time (s)
CNN-ELM	7	100.00	99.93	100.00	99.56	0.02
CNN-SVM	7	100.00	100.00	98.45	98.46	0.09
CNN-Softmax	5	99.63	99.56	100.00	98.25	1.89

## Data Availability

The data presented in this study are available on request from the corresponding author.

## References

[1] Zheng X.C., Li Y.Y., Wei W.S., Peng Y.K. (2019). Detection of adulteration with duck meat in minced lamb meat by using visible near-infrared hyperspectral imaging. Meat Sci..

[2] Lopez-Maestresalas A., Insausti K., Jaren C., Perez-Roncal C., Urrutia O., Beriain M.J., Arazuri S. (2019). Detection of minced lamb and beef fraud using NIR spectroscopy. Food Control.

[3] Leng T., Li F., Xiong L.A., Xiong Q., Zhu M.T., Chen Y. (2020). Quantitative detection of binary and ternary adulteration of minced beef meat with pork and duck meat by NIR combined with chemometrics. Food Control.

[4] Sorensen K.M., Khakimov B., Engelsen S.B. (2016). The use of rapid spectroscopic screening methods to detect adulteration of food raw materials and ingredients. Curr. Opin. Food Sci..

[5] Kamal M., Karoui R. (2015). Analytical methods coupled with chemometric tools for determining the authenticity and detecting the adulteration of dairy products: A review. Trends Food Sci. Technol..

[6] Oliveira M.M., Cruz-Tirado J.P., Barbin D.F. (2019). Nontargeted Analytical Methods as a Powerful Tool for the Authentication of Spices and Herbs: A Review. Compr. Rev. Food Sci. Food Saf..

[7] Bhat M.M., Salahuddin M., Mantoo I.A., Adil S., Jalal H., Pal M.A. (2016). Species-specific identification of adulteration in cooked mutton Rista (a Kashmiri Wazwan cuisine product) with beef and buffalo meat through multiplex polymerase chain reaction. Vet. World.

[8] Liu W.W., Wang X.N., Tao J., Xi B.S., Xue M., Sun W.P. (2019). A Multiplex PCR Assay Mediated by Universal Primers for the Detection of Adulterated Meat in Mutton. J. Food Prot..

[9] Wang Q., Li L., Ding W., Zhang D.Q., Wang J.Y., Reed K., Zhang B.C. (2019). Adulterant identification in mutton by electronic nose and gas chromatography-mass spectrometer. Food Control.

[10] Kucha C.T., Liu L., Ngadi M., Gariepy C. (2021). Anisotropic effect on the predictability of intramuscular fat content in pork by hyperspectral imaging and chemometrics. Meat Sci..

[11] Silva L.C.R., Folli G.S., Santos L.P., Barros I.H.A.S., Oliveira B.G., Borghi F.T., dos Santos F.D., Filgueiras P.R., Romao W. (2020). Quantification of beef, pork, and chicken in ground meat using a portable NIR spectrometer. Vib. Spectrosc..

[12] Wold J.P., O’Farrell M., Tschudi J., Eskildsen C.E., Andersen P.V., Ottestad S. (2020). In-line and non-destructive monitoring of core temperature in sausages during industrial heat treatment by NIR interaction spectroscopy. J. Food Eng..

[13] Bailes K.L., Meyer R.G., Piltz J.W. (2022). Prediction of the intramuscular fat and protein content of freeze-dried ground meat from cattle and sheep using near-infrared spectroscopy (NIRS). Int. J. Food Sci. Technol..

[14] Li Q.L., Wu X.H., Zheng J., Wu B., Jian H., Sun C.Z., Tang Y.B. (2022). Determination of Pork Meat Storage Time Using Near-Infrared Spectroscopy Combined with Fuzzy Clustering Algorithms. Foods.

[15] Wei C., Li X. (2022). A near-infrared spectroscopy method for the detection of texture profile analysis of Litopeneo vannamei based on partial least squares regression. J. Food Process. Eng..

[16] Barragán W., Aalhus J.L., Penner G., Dugan M.E.R., Juárez M., López-Campos Ó., Vahmani P., Segura J., Angulo J., Prieto N. (2021). Authentication of barley-finished beef using visible and near infrared spectroscopy (Vis-NIRS) and different discrimination approaches. Meat Sci..

[17] Alamprese C., Amigo J.M., Casiraghi E., Engelsen S.B. (2016). Identification and quantification of turkey meat adulteration in fresh, frozen-thawed and cooked minced beef by FT-NIR spectroscopy and chemometrics. Meat Sci..

[18] Boiret M., Chauchard F. (2017). Use of near-infrared spectroscopy and multipoint measurements for quality control of pharmaceutical drug products. Anal. Bioanal. Chem..

[19] Duan H.W., Zhu R.G., Wang L., Xu W.D., Ma B.X. (2016). Effects of Regions of Interest (ROIs) on Detection Models of Mutton pH Based on Hyperspectral Imaging. Spectrosc. Spect. Anal..

[20] Song L., Chen E., Zheng T., Li J., Wang H., Zhu X. (2022). Blended fabric with integrated neural network based on attention mechanism qualitative identification method of near infrared spectroscopy. Spectrochim. Acta A.

[21] Ravichandran P., Viswanathan S., Ravichandran S., Pan Y.J., Chang Y.K. (2022). Estimation of grain quality parameters in rice for high-throughput screening with near-infrared spectroscopy and deep learning. Cereal Chem..

[22] Chen X., Cheng G., Liu S., Meng S., Jiao Y., Zhang W., Xu J. (2022). Probing 1D convolutional neural network adapted to near-infrared spectroscopy for efficient classification of mixed fish. Spectrochim. Acta A.

[23] Padarian J., Minasny B., McBratney A.B. (2019). Using deep learning to predict soil properties from regional spectral data. Geoderma Reg..

[24] Chen Y.Y., Wang Z.B. (2019). Feature selection based convolutional neural network pruning and its application in calibration modeling for NIR spectroscopy. Chemom. Intell. Lab. Syst..

[25] Li L.Q., Pan X.P., Feng Y.C., Yin L.H., Hu C.Q., Yang H.H. (2019). Deep Convolution Network Application in Identification of Multi-Variety and Multi-Manufacturer Pharmaceutical. Spectrosc. Spect. Anal..

[26] Su T., Mu S., Shi A., Cao Z., Dong M. (2019). A Cnn-Lsvm Model for Imbalanced Images Identification of Wheat Leaf. Neural Netw. World.

[27] Sharma J., Granmo O.C., Goodwin M. (2018). Deep CNN-ELM Hybrid Models for Fire Detection in Images. Artificial Neural Networks and Machine Learning—ICANN 4 October 2018, Pt Ⅲ.

[28] Dixit Y., Casado-Gavalda M.P., Cama-Moncunill R., Cama-Moncunill X., Cullen P.J., Sullivan C. (2016). Prediction of beef fat content simultaneously under static and motion conditions using near infrared spectroscopy. J. Near Infrared Spectrosc..

[29] Dixit Y., Casado-Gavalda M.P., Cama-Moncunill R., Cullen P.J., Sullivan C. (2017). Challenges in Model Development for Meat Composition Using Multipoint NIR Spectroscopy from At-Line to In-Line Monitoring. J. Food Sci..

[30] Cortes V., Cubero S., Blasco J., Aleixos N., Talens P. (2019). In-line Application of Visible and Near-Infrared Diffuse Reflectance Spectroscopy to Identify Apple Varieties. Food Bioprocess. Technol..

[31] Galvao R.K., Araujo M.C., Jose G.E., Pontes M.J., Silva E.C., Saldanha T.C. (2005). A method for calibration and validation subset partitioning. Talanta.

[32] Gong W.F., Chen H., Zhang Z.H., Zhang M.L., Wang R.H., Guan C., Wang Q. (2019). A Novel Deep Learning Method for Intelligent Fault Diagnosis of Rotating Machinery Based on Improved CNN-SVM and Multichannel Data Fusion. Sensors.

[33] Dyrmann M., Karstoft H., Midtiby H.S. (2016). Plant species classification using deep convolutional neural network. Biosyst. Eng..

[34] Nasiri A., Omid M., Taheri-Garavand A. (2020). An automatic sorting system for unwashed eggs using deep learning. J. Food Eng..

[35] Zhao H.T., Feng Y.Z., Chen W., Jia G.F. (2019). Application of invasive weed optimization and least square support vector machine for prediction of beef adulteration with spoiled beef based on visible near-infrared (Vis-NIR) hyperspectral imaging. Meat Sci..

[36] Kuswandi B., Cendekiawan K.A., Kristiningrum N., Ahmad M. (2015). Pork adulteration in commercial meatballs determined by chemometric analysis of NIR Spectra. J. Food Meas. Charact..

[37] Morsy N., Sun D.W. (2013). Robust linear and non-linear models of NIR spectroscopy for detection and quantification of adulterants in fresh and frozen-thawed minced beef. Meat Sci..

[38] Pu H.B., Kamruzzaman M., Sun D.W. (2015). Selection of feature wavelengths for developing multispectral imaging systems for quality, safety and authenticity of muscle foods—A review. Trends Food Sci. Technol..

